# From MEK inhibition to rational combination strategies for NF1-associated plexiform neurofibromas

**DOI:** 10.3389/fonc.2026.1976580

**Published:** 2026-10-30

**Authors:** Shuhan Diao, Haiyan Qin, Jiao Kong, Lianbo Zhang

**Affiliations:** Department of Plastic Surgery, China-Japan Union Hospital of Jilin University, Changchun, China

**Keywords:** combination therapy, MEK inhibitor, neurofibromatosis type 1, plexiform neurofibroma, targeted therapy, tumor microenvironment

## Abstract

Plexiform neurofibromas (PNs) are among the most clinically burdensome tumor manifestations of neurofibromatosis type 1 (NF1) and can cause pain, disfigurement, neurological deficits, and compression of vital structures. Their diffuse growth along nerves and blood vessels often limits complete surgical resection. Biallelic *NF1* loss results in persistent activation of RAS–RAF–MEK–ERK signaling and provides a strong biological rationale for MEK inhibition. Selumetinib and mirdametinib have received regulatory approval in defined patient populations, while trametinib, luvometinib, and other MEK inhibitors have also demonstrated clinical activity. Nevertheless, responses remain predominantly partial. Some patients show primary nonresponse, limited tumor shrinkage, or treatment-limiting toxicity, and tumor regrowth may occur after dose reduction or treatment discontinuation. This review first summarizes molecular mechanisms and monotherapy evidence, then evaluates rational combinations and their clinical translation. These strategies include vertical inhibition of RAS–MAPK signaling, blockade of parallel survival pathways, targeting of residual tumor-cell states, and modulation of the tumor microenvironment. Combined MEK and SOS1 inhibition has relatively strong PN-specific *in vivo* support. KRAS–MEK inhibition has emerging mouse-model support, while selumetinib plus cabozantinib is undergoing clinical dose exploration. Strategies targeting CDKs or YAP/TEAD warrant further translational evaluation. In contrast, negative or borderline findings involving SHP2, mTOR, and several microenvironment-directed approaches demonstrate that biological plausibility does not necessarily translate into therapeutic benefit. Because NF1-associated PNs are predominantly benign tumors requiring long-term management, rational combination therapy should not simply increase treatment intensity. Instead, it should address a defined limitation of monotherapy and demonstrate a favorable net clinical benefit over an active single agent. Future treatment may therefore evolve toward mechanism-guided approaches incorporating reduced-dose combinations, sequential or intermittent therapy, rechallenge, and surgery after pharmacological tumor reduction. Tumor volume, pain, function, long-term safety, resectability, and disease control after treatment withdrawal should collectively inform therapeutic value.

## 1 Introduction

Neurofibromatosis type 1 (NF1) is an autosomal dominant tumor-predisposition syndrome caused by pathogenic variants in the NF1 tumor suppressor gene. Its highly heterogeneous phenotype includes pigmentary, skeletal, vascular, and neurodevelopmental abnormalities, together with benign and malignant tumors (1–5). Plexiform neurofibromas (PNs) are a characteristic neoplastic manifestation of NF1. They arise when the remaining functional *NF1* allele is inactivated in Schwann-lineage cells, producing biallelic *NF1* loss (2, 6–14). PNs are often congenital or develop in early childhood and extend diffusely along one or more nerve fascicles. Magnetic resonance imaging (MRI) studies show that internal lesions are common, although prevalence estimates vary with age, lesion definition, and ascertainment (2, 15–23). Their clinical consequences depend on anatomical location and growth pattern. PNs may cause chronic pain, disfigurement, motor impairment, and airway or spinal cord compression, with substantial effects on quality of life (2, 3, 15, 16, 22–29). New persistent pain, rapid focal enlargement, or progressive neurological deterioration should prompt evaluation for atypical neurofibromatous neoplasms or malignant peripheral nerve sheath tumors (MPNSTs) (2, 4, 5, 17, 19).

Complete resection remains the preferred treatment for localized PNs when achievable without unacceptable morbidity and offers the possibility of cure (2, 4). Surgery is particularly important for tumors causing neurological compromise or compression of vital structures. However, PNs often involve major nerves and blood vessels, have poorly defined margins, and are highly vascular. Resection can therefore cause substantial hemorrhage or permanent neurological impairment. When complete excision is unsafe, selective debulking may relieve compression or improve function, but residual tumor can regrow and necessitate further procedures (30, 31). Urgent decompression should not be delayed while awaiting a pharmacological response when progressive neurological deficits threaten irreversible functional loss (2). Conventional cytotoxic chemotherapy has no established efficacy against benign PNs. Radiotherapy likewise has no established routine role because evidence for durable benefit is limited and radiation-associated subsequent malignancies are a concern in NF1 (2). Its use in selected patients with confirmed MPNST represents a distinct clinical setting and should not be extrapolated to benign PNs (4). These limitations highlight the value of targeted systemic therapy for symptomatic tumors that cannot be completely resected without substantial morbidity. Targeted therapy should therefore complement, rather than universally replace, surgery within an individualized multidisciplinary treatment plan. Its goals should include symptom relief and functional preservation, alongside rehabilitation, pain management, and long-term surveillance (4, 5, 22, 23, 29, 32, 33).

The clinical success of mitogen-activated protein kinase kinase (MEK) inhibitors has transformed a disease that previously lacked reliable systemic therapy and has validated RAS–MAPK dependence as a therapeutically relevant vulnerability in NF1-associated PNs. Nevertheless, substantial residual tumor usually persists after treatment. Response depth, time to response, and long-term tolerability vary considerably among patients. Dermatologic, gastrointestinal, ocular, cardiac, or muscular toxicities may require dose modification, while reduced treatment intensity or discontinuation can be followed by renewed tumor growth (34–39). The central therapeutic question has therefore shifted from whether pharmacological tumor shrinkage is achievable to how deeper, more durable, and sustainable disease control can be obtained (40, 41).

Existing reviews have comprehensively summarized the clinical management of NF1-associated PNs, evidence for MEK inhibitors, and treatment pathways across pediatric and adult populations (2, 42). As several MEK inhibitors have demonstrated clinical activity, further comparisons based solely on drug class or objective response rate are unlikely to resolve the major translational questions facing the field. More relevant questions include which limitations of monotherapy have an actionable biological basis, which patients may require an additional therapeutic intervention, and what level of evidence should be required before a candidate combination advances from experimental models into clinical testing.

Building on current management reviews (2, 42), this review links distinct patterns of inadequate response to monotherapy with corresponding therapeutic strategies. We evaluate vertical inhibition of RAS–MAPK signaling, blockade of parallel survival pathways, targeting of residual tumor-cell states, and modulation of the tumor microenvironment. Evidence from human PN studies is distinguished from PN-specific preclinical evidence and indirect findings derived from MPNSTs or other NF1/RAS-driven tumors. Negative studies are also considered when assessing the translational priority of candidate combinations. Finally, we discuss patient selection, treatment scheduling, biomarkers, integration with surgery, and clinically meaningful trial endpoints to provide a therapeutic development framework suited to the long-term management of NF1-associated PNs.

## 2 Literature search strategy

This narrative review used PubMed/MEDLINE as the principal source of peer-reviewed literature. Searches combined “neurofibromatosis type 1” or “plexiform neurofibroma” with drug names, treatment modalities, and pathway terms. Additional concepts included natural history, surgery, volumetric MRI, patient-reported outcomes, Schwann cells, macrophages, fibroblasts, extracellular matrix, mechanotransduction, tissue stiffness, RAS–MAPK, PI3K–AKT–mTOR, RAC1, SHP2, SOS1, CDK, YAP/TEAD, HSP90, PP2A, valosin-containing protein, and tumor microenvironment. Clinical evidence was preferentially derived from prospective trials, randomized studies, and long-term follow-up studies conducted directly in patients with NF1-associated PNs. Because human data on combination therapy remain limited, genetically engineered animal models, patient-derived models, and three-dimensional cultures with direct relevance to PN biology were also considered. Findings from MPNSTs and other NF1- or RAS-driven tumors were used only to support mechanistic discussion and were not directly extrapolated to the therapeutic efficacy of benign PNs. ClinicalTrials.gov, publicly available regulatory information, and recent conference abstracts directly relevant to PN drug development were additionally reviewed. Literature coverage extends through October 12, 2026. Reporting quality was cross-checked against the Scale for the Assessment of Narrative Review Articles (SANRA) (43). This was a narrative, not systematic, review; no protocol was registered, and no formal risk-of-bias assessment or quantitative evidence synthesis was performed.

### 2.1 Inclusion criteria

Eligible publications addressed NF1-associated PN biology, natural history, or treatment, including clinical studies and disease-relevant experimental models. Foundational mechanistic studies and peer-reviewed reviews were retained when directly relevant to the therapeutic rationale.

### 2.2 Exclusion criteria

Studies confined to cutaneous neurofibromas or NF2-related schwannomatosis were excluded from PN efficacy conclusions. Duplicate reports, conference-only efficacy data, and unverified treatment claims were not used as independent evidence. Findings from MPNSTs or other tumors were included only as explicitly identified mechanistic context.

## 3 Molecular pathogenesis and therapeutic targets in NF1-associated plexiform neurofibromas

### 3.1 Biallelic *NF1* loss and the RAS signaling network

Individuals with NF1 carry a germline loss-of-function alteration in one *NF1* allele. PN development generally requires somatic loss or pathogenic alteration of the remaining wild-type allele within the Schwann-cell lineage, resulting in biallelic *NF1* inactivation (6–8). Tumorigenic Schwann cells therefore lack functional neurofibromin, whereas most non-neoplastic cells within the tumor microenvironment remain heterozygous for *NF1*. This “second hit in tumor cells–heterozygous host microenvironment” architecture creates both cell-autonomous signaling abnormalities and marked dependence on the surrounding stromal environment (9, 10, 44).

Neurofibromin functions as a RAS GTPase-activating protein and promotes conversion of RAS-GTP to RAS-GDP. Loss of neurofibromin increases or prolongs active RAS-GTP, thereby enhancing RAF–MEK–ERK signaling. ERK regulates cell-cycle entry, survival, migration, and differentiation through transcriptional and phosphorylation-dependent mechanisms and represents one of the best-established effector pathways in NF1-associated PNs (46–48).

RAS-GTP promotes membrane recruitment and dimerization of RAF-family kinases, including ARAF, BRAF, and CRAF (46, 49). RAF phosphorylates the MEK1/2 activation loop, enabling MEK-dependent dual phosphorylation of ERK1/2. NF1 loss impairs GAP-stimulated RAS hydrolysis rather than abolishing intrinsic RAS GTPase activity; a dominant RAF isoform has not been established in human PNs (46, 47, 49).

RAS also activates several downstream branches, including PI3K–AKT–mTOR, Ral guanine nucleotide exchange factors, and RAC. The PI3K–AKT–mTOR pathway regulates protein synthesis, metabolism, and cellular growth and is influenced by the neurofibromin–TSC2 network (50). *NF1* loss therefore generates a signaling network characterized by feedback regulation and pathway crosstalk rather than activation of a single linear cascade (51).

RAS activity also remains influenced by upstream signaling. Following activation of receptor tyrosine kinases (RTKs), adaptor proteins and the SH2 domain-containing protein tyrosine phosphatase 2 (SHP2) facilitate Son of Sevenless 1 (SOS1)-mediated GDP–GTP exchange. Loss of neurofibromin impairs the inactivation of RAS-GTP but does not permanently lock RAS in its GTP-bound state. RAS retains intrinsic GTPase activity and, after returning to the GDP-bound state, may be reactivated through SOS1. RTKs, SHP2, and SOS1 therefore remain potentially druggable upstream nodes despite *NF1* loss (46, 49, 52, 53).

*NF1* loss also affects cell-cycle regulation, protein homeostasis, and mechanotransduction. RAS–ERK signaling can promote activation of cyclin-dependent kinases (CDKs), whereas heat shock protein 90 (HSP90) maintains the folding and stability of multiple signaling proteins. Dysregulated Hippo signaling can promote nuclear translocation of YAP/TAZ and TEAD-dependent transcription of genes involved in proliferation and survival. Protein phosphatase 2A (PP2A) negatively regulates several pathways, including MAPK, AKT–mTOR, Wnt, and Hippo signaling, through substrate dephosphorylation. These nodes represent potential therapeutic vulnerabilities beyond MEK, although their disease specificity, consistency across patients, and suitability for chronic pharmacological intervention remain incompletely defined (45, 54–59).

### 3.2 Schwann-cell states, stromal heterogeneity, and the tumor microenvironment

The tumor-initiating compartment of PNs arises within the *NF1-deficient* Schwann-cell lineage, but PNs are not composed of a single Schwann-cell state. Schwann cells and their precursors at different stages of differentiation can produce distinct cytokine, chemokine, and extracellular matrix (ECM) programs and can remodel surrounding neural tissue. Single-cell and lineage studies have shown that multiple *NF1-deficient* Schwann-cell populations interact with fibroblasts, immune cells, and neural structures to form a mutually supportive ecosystem (60). Consequently, distinct lesions—and regions within a lesion—may plausibly differ in cellular state and microenvironmental context, with potential consequences for MAPK dependence and drug exposure (10–14, 61–63).

Myeloid cells represent a major component of the PN microenvironment. Macrophages may account for a substantial proportion of cells within some neurofibromas, and their origin, phenotype, and function vary according to disease stage and local signaling conditions. Colony-stimulating factor 1 (CSF1)–CSF1 receptor (CSF1R), complement C5a–C5a receptor (C5aR), STAT3, and SHP2 signaling have all been implicated in monocyte recruitment or macrophage-state maintenance (52, 64–66). Mast cells may be recruited by KIT ligand secreted from *NF1-deficient* Schwann cells and participate in tissue remodeling through release of growth factors and proteases. T cells are less abundant but may indirectly influence neurofibroma growth by modifying myeloid-cell behavior. These immune populations should not be regarded as uniformly or permanently protumorigenic. Their functional effects may vary over time, and microenvironment-directed treatment therefore requires consideration of both cellular state and disease stage (44, 67–69).

Fibroblasts and the ECM form another supportive component of the PN microenvironment. Human and murine neurofibromas contain abundant collagen, laminin, and other basement membrane proteins. Transforming growth factor β1 from immune cells can promote deposition of these proteins by Schwann cells and may alter tissue stiffness, cellular adhesion, and drug diffusion (70). Three-dimensional coculture models further suggest that fibroblast-derived paracrine signals can reduce the sensitivity of PN cells to MEK inhibition and increase P-glycoprotein-mediated drug efflux (71). In a patient-derived three-dimensional culture model, increased ECM stiffness promoted PN-cell growth and P-glycoprotein expression and was associated with reduced selumetinib sensitivity (72). Limited tumor regression may therefore reflect not only tumor-cell intrinsic resistance but also stromal protection and heterogeneous intratumoral drug exposure (62, 73, 74).

Together, these tumor-cell-intrinsic and microenvironmental processes form an integrated signaling ecosystem that supports PN initiation, growth, and persistence (**Figure 1**).

**Figure 1:**
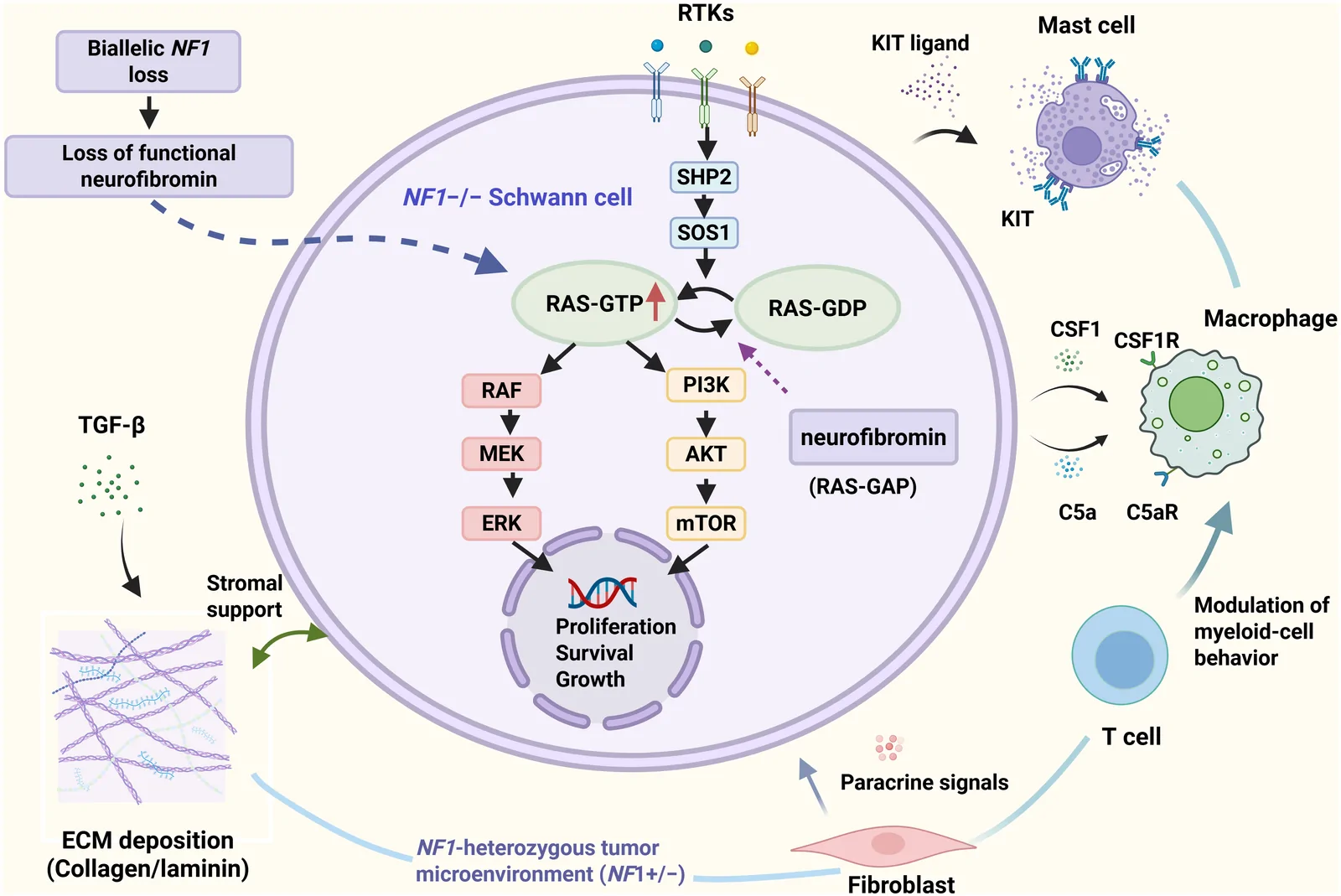
Molecular pathogenesis and microenvironmental support in NF1-associated plexiform neurofibromas. Biallelic *NF1* inactivation in Schwann-lineage tumor cells causes loss of functional neurofibromin, impairs RAS-GTP hydrolysis, and increases the active RAS-GTP pool. Receptor tyrosine kinase inputs through SHP2 and SOS1 can further sustain RAS cycling. Active RAS stimulates the RAF–MEK–ERK and PI3K–AKT–mTOR pathways, thereby promoting tumor-cell proliferation, survival, and growth. The NF1-heterozygous tumor microenvironment provides additional support through interactions among immune and stromal cells. KIT ligand–KIT signaling promotes mast-cell recruitment, whereas CSF1–CSF1R and C5a–C5aR signaling contribute to macrophage recruitment and state maintenance. T cells may modify myeloid-cell behavior, while fibroblast-derived paracrine signals provide additional stromal support. TGF-β-dependent signaling promotes extracellular-matrix deposition, including collagen and laminin, which may influence tissue mechanics and treatment response. The schematic highlights selected interactions and does not encompass all cellular states or signaling pathways implicated in plexiform neurofibromas. Created in BioRender. Diao, S. (2026) https://BioRender.com/30xjrjm.

## 4 Current monotherapy and its limitations

### 4.1 MEK inhibitors

MEK inhibitors are small molecules that bind allosteric sites of MEK1/2 and reduce MEK-dependent phosphorylation of ERK1/2. They thereby suppress proliferation and growth-associated transcription in *NF1*-deficient Schwann cells and may also indirectly alter the ECM and myeloid-cell compartment (34, 48, 64, 70, 75).

Radiological response in PN trials is generally assessed using semiautomated three-dimensional volumetric MRI. A confirmed partial response is typically defined as at least a 20% reduction in target PN volume from baseline on serial evaluation (76). This volumetric approach is more appropriate than conventional unidimensional criteria for PNs because of their irregular morphology and relatively slow growth (18, 77).

MEK inhibitors have become the principal class of systemic therapy for symptomatic, inoperable NF1-associated PNs (2, 42). However, direct comparison of objective response rates (ORRs) across studies is inappropriate because confirmation windows, follow-up duration, eligibility criteria, dosing, and imaging review procedures differ. Treatment schedule, long-term tolerability, pain and functional improvement, and the ability to maintain effective exposure are also clinically important. Representative monotherapy studies and their clinical positioning are summarized in **Table 1**.

**Table 1:** Published systemic monotherapy studies in NF1-associated plexiform neurofibromas by trial phase, with participant numbers, dosing, follow-up or treatment exposure, and clinical outcomes.

Study and phase	Participants and design	Oral dose and schedule	Follow-up, exposure or assessment window	Tumor outcome	Clinical interpretation
Phase I Luvometinib (93)	19 children 10/9 per dose	4 or 5 mg/m² QD	28-day cycles until PD/toxicity; median FU NV	Responses: 0/10 at 4 mg/m²; 6/9 at 5 mg/m²	Pain benefit in a small subset; nonrandomized dose cohorts.
Phase I/II follow-up Selumetinib (35)	74 children Pooled cohorts	Phase I escalation; phase II 25 mg/m² BID	Median Tx 57.5 cycles; up to 5 additional years of observation	ORR 70%; 44/74 responses lasted ≥12 cycles	Sustained pain benefit; late toxicities and regrowth after stopping.
Phase II SPRINT (34)	50 children Single-arm	Selumetinib 25 mg/m² BID	Median Tx 36 cycles	ORR 68%	Pain, function and QoL improved; no concurrent control.
Phase II NCI adult study (36)	33 adults Single-arm	Selumetinib 50 mg BID; first 2 started at 75 mg BID	Median Tx 28 cycles	ORR 63.6%; median maximal volume reduction 23.6%	Pain intensity/interference improved; delayed responses occurred.
Phase II NF106 (89)	19 aged ≥16 y Single-arm	Mirdametinib 2 mg/m² BID (max 4 mg/dose), days 1–21	Assessments at cycles 4, 8, 12, 18 and 24	ORR 42% by cycle 12	Pain improved; movement/balance benefit in responders.
Phase II TRAIN (90)	30 adults Single-arm	Trametinib 2 mg QD	Median FU 24 mo; median Tx 15 mo	ORR 47% at cycle 12; 50% at any time	Pain improved; QoL unchanged; 40% stopped for AEs.
Phase II TRAM-01 (39)	46 children/young adults Single-arm	Trametinib 0.032 (<6 y) or 0.025 (≥6 y) mg/kg QD; max 2 mg	Median FU 45.7 mo; Tx ≤18 cycles	ORR 47.8%	QoL improved over time, not baseline-to-end; subsequent treatment sometimes needed.
Phase II Cabozantinib (96)	19 evaluable Age ≥16 y; single-arm	40 mg QD; increase to 60 mg QD if tolerated	Response assessment at cycle 12; Tx ≤24 cycles	ORR 42.1% (8/19)	Pain improved in responders; global QoL unchanged; multikinase toxicity.
Phase II Imatinib (97)	36 enrolled 23 completed 6 mo	220 mg/m² BID (children); 400 mg BID (adults)	6-mo treatment/primary assessment	ORR 17% (6/36 ITT); 26% (6/23 completers)	Benefit in a subset; high attrition and short treatment.
Phase II Sirolimus (101)	49 enrolled; 46 efficacy-evaluable Historical control	Start 0.8 mg/m² BID; trough target 10–15 ng/mL	Serial MRI for TTP; median FU NV	Median TTP 15.4 vs 11.9 mo (historical control)	Modest growth delay; no significant pain or overall QoL improvement.
Phase IIa Tunlametinib (95)	15 adults Single-arm	9 mg BID; continuous 21-day cycles	Median FU/Tx NV	ORR 53.3%	Several patient-reported outcomes improved; small cohort.
Phase IIb ReNeu (38)	58 adults/56 children Single-arm	Mirdametinib 2 mg/m² BID (max 4 mg/dose), days 1–21	24-cycle primary window; median Tx 21.8/22.0 mo	ORR 41%/52% (adults/children)	Pain and QoL improved; unblinded symptom assessment.
Phase III KOMET (37)	145 randomized adults 71 active/74 placebo	Selumetinib 25 mg/m² BID vs placebo	ORR by cycle 16; symptoms at cycle 12; crossover allowed	ORR 20% vs 5% by cycle 16	Pain/QoL between-group differences not significant; crossover limits long-term comparison.

FU, actual follow-up; Tx, treatment exposure; assessment windows are distinct from FU. NV, not verifiable from the accessible report (not inferred). Cycles are 28 days except tunlametinib (21 days). Mirdametinib: 21 days on/7 days off. SPRINT and pooled follow-up share participants (34, 35). ORRs are not directly comparable across trials.

Phase colors: I, blue; I/II, blue-gray; II/IIa/IIb, amber; III, purple. No dedicated neoadjuvant trial is included. AE, adverse event; BID/QD, twice/once daily; ITT, intention to treat; mo, months; ORR, objective response rate; PD, progression; QoL, quality of life; TTP, time to progression; y, years.

#### 4.1.1 Selumetinib

Selumetinib is an oral, selective allosteric MEK1/2 inhibitor generally administered twice daily according to body surface area. In a pediatric phase I study, 17 of 24 patients (71%) achieved a confirmed partial response, establishing 25 mg/m² twice daily as the dose taken forward for subsequent development (75). The phase II SPRINT study enrolled 50 children with inoperable PNs associated with clinically relevant morbidity. Thirty-four patients (68%) achieved a confirmed response, accompanied by improvements in pain, function, and quality of life, demonstrating that volumetric changes can translate into clinically meaningful benefit (34).

Long-term follow-up of 74 patients treated in the phase I/II program showed a confirmed ORR of 70% after a median of 57.5 treatment cycles; 44 of 74 patients (59%) had responses lasting at least 12 cycles (35). Pain improvement was generally maintained, although tumor regrowth occurred in some patients after dose reduction or treatment discontinuation. Some recognized adverse events also first emerged after several years of therapy, emphasizing the need for continued cardiac, ophthalmologic, creatine kinase, and dermatologic monitoring despite early tolerability (41, 78). A separate analysis of 98 pediatric participants with a median follow-up of 5.1 years found no apparent clinically significant hematologic changes associated with selumetinib (79).

A 2026 meta-analysis of 23 studies involving 769 patients estimated a pooled objective response rate of 56% (95% CI, 46–65%), a disease-control rate of 96% (95% CI, 91–98%), and a grade ≥3 adverse-event rate of 13% (95% CI, 6–25%). Response estimates were heterogeneous, and the pooled progression estimate described outcomes during treatment rather than durability after withdrawal (80). A separate meta-analysis of seven trials found statistically significant reductions in pain intensity and pain interference during MEK-inhibitor therapy. However, the mean population-level change in pain intensity did not reach the prespecified threshold for clinical importance, whereas responder analyses indicated clinically meaningful improvement in a subset of patients (81).

Adult evidence includes complementary single-arm and randomized studies. A National Cancer Institute (NCI) phase II study enrolled 33 adults and reported an ORR of 63.6%, with a median maximal target-PN volume reduction of 23.6%. No patient had progressed relative to baseline at the data cutoff, and both pain intensity and pain interference decreased (36). Paired biopsies showed marked suppression of ERK1/2 phosphorylation, whereas consistent compensatory AKT phosphorylation was not observed at the predefined sampling time points.

The phase III KOMET study randomly assigned 145 adults to selumetinib or placebo. Confirmed ORRs by cycle 16 were 20% and 5%, respectively (*p* = 0.011). Among patients with baseline chronic pain scores of at least 3, pain outcomes numerically favored selumetinib, although the between-group difference was not statistically significant, and PN-specific quality-of-life scores did not differ significantly (37). The difference in ORR between the NCI phase II study and KOMET may reflect differences in observation windows and study design and should not be interpreted as contradictory estimates of drug activity (82).

KOMET permitted placebo crossover at progression or after cycle 12, limiting longer-term randomized comparisons. Its fixed response window may also miss delayed responses, while volumetric efficacy should be distinguished from the less conclusive comparative symptom outcomes (37).

Common class-related toxicities of selumetinib include acneiform dermatitis, paronychia, diarrhea, elevated creatine phosphokinase, reduced left ventricular ejection fraction, and ocular events. In 2025, the US Food and Drug Administration (FDA) expanded the indication to adults and to pediatric patients aged ≥1 year with NF1 and symptomatic, inoperable PNs. In the phase I/II SPRINKLE study, a granule formulation produced exposure comparable to the capsule formulation and had a manageable safety profile in children aged 1 to <7 years (83). Prospective studies in Chinese and Korean patients have further supported activity across populations (84, 85). A small single-center Chinese pediatric cohort reported a confirmed ORR of 81.3% among 16 participants after a median of 45 cycles, although the sample size limits generalizability (86). Overall, selumetinib has the most mature clinical evidence and broad treatment experience, although continuous twice-daily administration and long-term monitoring remain important burdens (40, 41, 78).

For children, prolonged treatment additionally requires longitudinal assessment of growth, pubertal development, and neurodevelopmental function (78, 85). Body surface area should be reassessed during growth, with toxicity-specific treatment interruption and dose reduction when indicated (78). Uncontrolled reports of cognitive or growth improvements do not establish long-term developmental safety (85). Preschool children, school-age children, and adolescents may also require different formulations and outcome assessments (42, 83).

Recent prospective and pharmacokinetic evidence further informs long-term management. In a 23-patient prospective case series with a mean follow-up of 1,085 days, 13 patients (57%) achieved more than 20% tumor-volume reduction, seven (30%) had stable tumor volume, and three (13%) had tumor growth; no severe adverse events were reported (87). A single-center pilot therapeutic drug-monitoring study found higher dose-normalized selumetinib exposure among children with adverse events than among those without toxicity. These preliminary findings support further evaluation of exposure-guided dosing but do not establish a validated therapeutic range or an efficacy-based dosing standard (88).

#### 4.1.2 Mirdametinib

Mirdametinib is also an oral allosteric MEK1/2 inhibitor and is administered on an intermittent schedule of 3 weeks on treatment followed by 1 week off in each 28-day cycle. The phase II NF106 trial enrolled 19 adolescents and adults with symptomatic or progressive inoperable PNs. By cycle 12, 8 patients (42%) had achieved partial responses, and pain intensity decreased among evaluable patients. Improvements in movement and balance were reported in responders (89).

The pivotal phase IIb ReNeu study enrolled 58 adults and 56 children aged 2–17 years and used blinded independent central review. Confirmed ORRs were 41% in adults and 52% in children, with median best target-PN reductions of 41% and 42%, respectively (38). Improvements in pain, pain interference, and health-related quality of life emerged early during therapy and were maintained during follow-up.

Blinded imaging review strengthens response assessment but does not remove bias from the single-arm design. Unblinded symptom reporting and incomplete follow-up also limit causal interpretation, and the separate age cohorts cannot establish an age-dependent treatment effect (38).

The FDA approved mirdametinib in 2025 for patients aged ≥2 years with symptomatic NF1-associated PNs not amenable to complete resection. Distinguishing features include intermittent administration, no fasting requirement, and a pediatric-compatible oral formulation. Although intermittent dosing may facilitate long-term treatment for some patients, current evidence does not establish that it is less toxic over the long term than all continuous MEK-inhibitor regimens. Cross-trial efficacy comparisons are also limited by the absence of a concurrent control group in ReNeu (78).

#### 4.1.3 Trametinib

Trametinib is a once-daily MEK1/2 inhibitor. The phase II TRAIN study enrolled 30 adults with symptomatic, inoperable PNs and administered trametinib at 2 mg once daily. Fourteen patients (47%) achieved partial responses by cycle 12, and 15 (50%) responded at any time during treatment. Median best volumetric reduction was 22%, and the median time to best response was 12 cycles (90). Pain and pain interference improved, whereas overall quality of life did not change substantially.

The most notable limitation of this study was tolerability. Forty percent of participants discontinued treatment because of adverse events, including serious events such as pneumonia and rhabdomyolysis. A dedicated cardiac safety analysis found asymptomatic, reversible cancer therapy–related cardiac dysfunction in 7 of 30 adults; one participant permanently discontinued trametinib (91). Thus, agents targeting the same pathway may produce broadly similar volumetric activity but substantially different sustainable therapeutic indices (90).

The prospective TRAM-01 study enrolled 46 children and young adults and treated patients for up to 18 cycles of 28 days. The ORR was 47.8%, with a median tumor-volume change of −19.3% (39). After completion of the fixed treatment period, 15 patients (32.6%) required further PN-directed intervention, with a median time of 10 months to treatment reinitiation. These findings underscore that disease control during therapy and durable control after treatment withdrawal are distinct clinical outcomes.

#### 4.1.4 Other MEK inhibitors

Luvometinib (FCN-159) is a potent, selective, once-daily MEK1/2 inhibitor. A phase I study in 19 adults supported 8 mg for further development based on pharmacodynamic and tumor-response data, although higher doses were associated with more grade ≥3 treatment-related adverse events (92). A pediatric phase I study also enrolled 19 patients. Six of nine patients in the 5 mg/m² group achieved confirmed responses, whereas none of ten patients in the 4 mg/m² group responded; one patient discontinued treatment because of rhabdomyolysis (93). These small nonrandomized dose cohorts do not establish a definitive exposure–response relationship or the width of the therapeutic window. In May 2025, China’s National Medical Products Administration approved luvometinib for children and adolescents aged ≥2 years with NF1 and symptomatic, inoperable PNs (94).

Tunlametinib (HL-085) produced a confirmed ORR of 53.3% in a single-arm phase IIa study of 15 adults, with a median tumor-volume reduction of 23.5% (95). In general, the potential advantages of newer MEK inhibitors lie in formulation, pharmacokinetics, and tolerability rather than an assumption that they will eliminate residual disease or long-term treatment dependence common to MEK inhibition.

#### 4.1.5 Common lessons from MEK-inhibitor studies

Despite variation in reported ORRs, MEK-inhibitor studies show a consistent pattern. PN shrinkage occurs across agents and age groups, supporting RAS–MAPK dependence as a clinically relevant vulnerability (34, 36–38, 40, 41, 48, 90). Complete responses remain rare, and most patients retain substantial residual tumor (34, 36–38, 90). Durable disease control often depends on continued drug exposure (35, 39). Regrowth after treatment cessation may reflect loss of pharmacological suppression. It should not, by itself, be classified as acquired resistance (35, 39).

Together, these findings establish MEK inhibitors as the benchmark for systemic PN therapy, but important limitations remain. Most responses are incomplete, and sustained control often requires prolonged drug exposure. Long-term treatment also creates cumulative toxicity and monitoring burdens (34–41, 78, 90).

### 4.2 Receptor tyrosine kinase inhibitors

RTK inhibitors may reduce growth-factor signaling to both tumor and stromal cells and thereby indirectly suppress RAS or PI3K signaling. Cabozantinib currently has the strongest clinical evidence among agents in this class for PNs. It inhibits multiple kinases, including MET, VEGFR2, AXL, RET, and KIT, and may affect tumor cells, vasculature, and the microenvironment simultaneously (96, 97).

A phase II study enrolled patients aged ≥16 years with progressive or symptomatic inoperable PNs. Eight of 19 evaluable patients (42.1%) achieved partial responses. Responders reported reductions in pain intensity and pain interference, without improvement in global quality of life (96). Gastrointestinal toxicity, fatigue, palmar–plantar erythrodysesthesia, hypertension, and hypothyroidism require active management. The small nonrandomized cohort does not establish equivalence with MEK inhibitors, but it demonstrates that non-MEK, multicompartment targeting can produce clinically relevant PN shrinkage.

Imatinib inhibits KIT, PDGFR, and ABL and was developed for PNs largely on the basis of mast-cell–KIT biology. In a phase II study, 6 of 36 enrolled patients (17%) achieved at least a 20% decrease in target-PN volume in the intention-to-treat analysis; the corresponding rate was 6 of 23 patients (26%) among those who completed 6 months of therapy (97). High attrition, short treatment duration, and lack of a control group limit interpretation, although the findings suggest that targeting supportive signals from non-neoplastic cells may influence PN growth in a subset of patients (44, 67).

Broader kinase coverage has not reliably improved PN outcomes. In a pediatric phase I study, sorafenib caused frequent dose-limiting toxicity and produced no objective responses (98). Evidence for sunitinib and nilotinib remains limited to a PN mouse model and PN-derived cell cultures, respectively (99, 100). These findings show that broader target coverage does not necessarily translate into greater clinical benefit. For tumors requiring long-term treatment, multikinase therapy must balance biological activity, effective exposure, and chronic toxicity.

### 4.3 PI3K/AKT/mTOR pathway inhibitors

The PI3K–AKT–mTOR axis regulates growth, metabolism, and protein synthesis and represents an important parallel pathway downstream of RAS. Sirolimus and everolimus primarily inhibit mTORC1, but their clinical effects as monotherapy have been more consistent with slowed growth than substantial tumor regression (50, 101–104).

In a phase II study of sirolimus for progressive PNs, median time to progression was 15.4 months compared with 11.9 months in a historical placebo cohort (101). This nonconcurrent comparison suggested modest disease stabilization but did not demonstrate reliable volumetric responses. In a separate nonprogressive PN cohort, no objective responses were observed after six courses of therapy (102). A phase IIa study of everolimus likewise showed no meaningful PN tumor-shrinking activity (103). A systematic review and meta-analysis of four small single-arm studies did not demonstrate a significant overall reduction in lesion size (104).

These findings do not exclude a biological role for mTOR signaling in PNs. Rather, they indicate that allosteric mTORC1 inhibition alone is insufficient to produce consistent tumor regression. mTORC2 can continue to support AKT signaling, while mTORC1 inhibition may relieve upstream feedback. Stomatitis, metabolic abnormalities, infection, cytopenias, and impaired wound healing further limit prolonged treatment. mTOR inhibitors are therefore not currently preferred as tumor-shrinking monotherapy for NF1-associated PNs (50, 101–104).

### 4.4 Other emerging monotherapy approaches

Earlier studies explored direct interference with RAS processing or broader biological therapies. The farnesyltransferase inhibitor tipifarnib failed to meet its prespecified primary endpoint in a randomized crossover study of 62 children and young adults with progressive PNs and did not generate convincing volumetric responses (105). Pegylated interferon α-2b produced responses in a minority of patients and may have prolonged time to progression, but systemic and neuropsychiatric toxicities limited its suitability for long-term therapy (106).

Other investigational monotherapies remain exploratory. A published phase I dose-escalation and pharmacokinetic report of pexidartinib (PLX3397; NCT02390752) included three participants with NF1-associated PNs; no PN response was documented, although two participants had stable disease. PN-specific efficacy has not been established (107). HLX-1502, an investigational mitochondrial dysregulator, is being evaluated in a recruiting phase II study (42), but its PN-specific activity and human exposure–response relationship remain to be defined.

High-throughput screening in PN-derived cell systems and computational integration with transcriptomic data from resected human PNs have identified potential vulnerabilities involving protein homeostasis, serotonin signaling, PI3K–AKT, and MAPK pathways (108, 109). The retinoid X receptor (RXR) agonist MSU-42011, the PP2A-modulating agent FTY720, and the SHP2 inhibitor RMC-4550 have shown biological effects in NF1-related models (52, 54, 110). A 2026 preclinical study identified valosin-containing protein (VCP) as another candidate vulnerability: genetic or pharmacological inhibition reduced growth in cellular and mouse models, but combining VCP and MEK inhibition did not improve efficacy beyond either single agent (111). These findings remain more useful for identifying candidate therapeutic nodes and pharmacodynamic biomarkers than for supporting routine monotherapy.

## 5 From monotherapy to mechanism-guided rational combination strategies

### 5.1 Limitations of monotherapy and the rationale for combination treatment

Suboptimal outcomes after MEK inhibition should not be grouped indiscriminately under the term “resistance.” Distinct clinical states include absence of meaningful volumetric response despite adequate drug exposure, partial response with substantial residual tumor, renewed progression during treatment, inability to maintain effective dosing because of toxicity, and tumor regrowth after dose reduction or discontinuation (19, 35, 39, 112).

These states have different therapeutic implications. Primary nonresponse should first prompt assessment of inadequate drug exposure, imaging uncertainty, or atypical or malignant transformation. Limited response depth may reflect persistent tumor-cell states, inadequate drug penetration, or stromal protection. Acquired biological adaptation is more appropriately considered when progression occurs despite sustained and adequate treatment exposure. Regrowth after withdrawal may simply indicate continued drug dependence and should not automatically be classified as acquired resistance (2, 4, 19, 20, 35, 39, 42, 112).

Candidate mechanisms can be grouped into pathway feedback, parallel survival signaling, altered tumor-cell states, and microenvironmental protection (55, 57, 71, 72, 112). Most supporting evidence remains preclinical. Acquired mutations are not established recurrent causes of MEK-inhibitor resistance in benign PNs, and increased pMEK alone does not demonstrate restored ERK activity (36, 112).

The rationale for combination therapy should therefore be based on a defined mechanism limiting monotherapy. Candidate combinations should demonstrate activity beyond an effective single agent at clinically achievable exposures (53, 66, 113). **Table 2** summarizes the evidence base, counterevidence, and translational priority of the principal candidate strategies.

**Table 2:** Candidate combinations for NF1-associated plexiform neurofibromas grouped by clinical or preclinical development stage, with mechanistic rationale, supporting evidence, limitations, and translational priority.

Stage and strategy	Main rationale	Evidence base	Main limitation or counterevidence	Translational priority
Clinical phase I Selumetinib + cabozantinib	Covers MAPK and multiple RTK/vascular/stromal signals	Dose escalation is recruiting (NCT06502171; planned n = 30). Dosing and assessment windows are summarized in **Table 3**.	Human combination efficacy data are unavailable.	Clinical dose-exploration stage
Preclinical MEK + SOS1	Restricts RAS reloading and ERK output	BI-3406 plus selumetinib outperformed MEK monotherapy in PN mice (53).	Human PN combination data are unavailable.	High preclinical priority
Preclinical MEK + KRAS	Restricts KRAS activation and downstream MEK signaling	BI6674 plus selumetinib enhanced tumor regression versus KRAS monotherapy in PN mice (114).	The MEK comparator pooled concurrent and historical controls; human efficacy and long-term tolerability remain unproven.	Promising preclinical priority
Preclinical MEK + SHP2	Restricts RTK–SOS1 input	SHP2 inhibition was active as monotherapy in PN models (52).	Concurrent MEK–SHP2 inhibition did not improve efficacy.	Low priority for unselected concurrent use
Preclinical MEK + AKT/mTOR	Blocks parallel survival/metabolic signaling	Adaptive pAKT was observed in PN cultures (112).	mTOR monotherapy showed weak activity, and indirect MPNST combination evidence was negative (101–104, 113).	Biomarker-selected exploratory priority
Preclinical MAPK + CDK	Targets residual cell-cycle dependence	Several PN models supported combined MAPK and CDK inhibition (55, 56).	Optimal partner selection and long-term toxicity remain uncertain.	Intermediate-to-high preclinical priority
Preclinical MEK + YAP/TEAD	Targets adaptive YAP transcriptional state	Verteporfin plus selumetinib enhanced activity in PN models (45, 57).	Available pharmacological tools have limited selectivity.	Early exploratory preclinical priority
Preclinical MEK + HSP90	Destabilizes multiple bypass signaling proteins	HSP90 inhibition enhanced selumetinib activity in PN cells and mice (58).	Chronic multiorgan toxicity may limit clinical use. The mouse study lacked an HSP90-inhibitor-alone arm.	Conditional preclinical priority
Preclinical MEK + PP2A modulation	Modulates PP2A-dependent signaling	FTY720 reduced tumor number in PN mice (54).	The combination did not clearly outperform monotherapy, and S1P1 effects confounded interpretation.	Early exploratory priority
Preclinical MEK + C5aR	Targets PN cells and myeloid support	MEK plus C5aR inhibition produced persistent microenvironmental changes (66).	The combination did not improve durable tumor control.	Low priority for shrinkage-directed development
Preclinical MEK + CSF1R/stromal targets	Reduces myeloid or ECM support	PN models show context-dependent effects of myeloid and stromal targeting (64, 69–74).	Effects depend on disease stage; combination benefit and long-term safety remain unproven.	Mechanistic and biomarker-development stage

Stage key: pale blue, clinical phase I; pale gray, preclinical strategies. Stage refers to the combination, not the approval status or phase of either drug alone. Translational priorities are qualitative assessments, not formal GRADE ratings or clinical recommendations. They consider PN specificity, active comparators, *in vivo* activity, feasibility, human evidence and long-term treatment (2, 42, 52–58, 64, 66, 70, 71, 112–114). ECM, extracellular matrix; PN, plexiform neurofibroma; RTK, receptor tyrosine kinase. Molecular targets are defined in the main text.

The cell colors in the “Stage and strategy” column indicate the development stage of each combination strategy: pale blue denotes clinical Phase I, and pale gray denotes preclinical investigation. These colors refer to the combination strategy, not to the approval status or development phase of either individual agent. They do not indicate efficacy, evidence quality, or translational priority.

### 5.2 Vertical targeting of the RAS–MAPK axis: upstream input and feedback reactivation

SOS1 is a guanine nucleotide exchange factor connecting RTKs to RAS. Although *NF1* loss delays RAS-GTP hydrolysis, it does not eliminate SOS1-dependent reactivation. Simultaneously restricting RAS nucleotide exchange and downstream MEK signaling therefore provides a biologically coherent form of vertical pathway inhibition (46, 49, 53).

In a Dhh-Cre–driven conditional *Nf1-knockout* PN model, the SOS1 inhibitor BI-3406 alone did not significantly reduce PN size. In contrast, exposure-guided BI-3406 plus selumetinib reduced tumor volume and proliferation more effectively than selumetinib alone. In some experiments, the selumetinib dose used in the combination group was also lower than that used for monotherapy (53). Because the study included an active single-agent comparator, *in vivo* tumor measurements, and exposure-informed dosing, it represents one of the stronger PN-specific preclinical combination datasets currently available. MEK–SOS1 inhibition can therefore be considered a relatively high-priority preclinical strategy, although long-term safety and optimal dose ratios must be established before clinical translation in PN.

Neurofibromin loss increases wild-type KRAS activity and downstream RAF–MEK–ERK signaling in NF1-associated plexiform neurofibromas. BI6674 (BI-3706674) is an oral KRAS inhibitor that blocks nucleotide exchange, retaining KRAS in its inactive GDP-bound state. Combining BI6674 with selumetinib targets upstream KRAS and downstream MEK, providing a rationale for more effective suppression of proliferative signaling. Their distinct effects on the tumor immune microenvironment provide an additional rationale for combination treatment. In a neurofibroma mouse model, this combination enhanced tumor regression compared with BI6674 alone. Tumor volume decreased in 13 of 16 mice receiving the combination. The selumetinib comparison pooled concurrent and historical controls, limiting conclusions about the added benefit over MEK monotherapy (114). However, these findings remain preclinical and do not establish reversal of clinical MEK inhibitor resistance. The durability of benefit and potential overlapping toxicities require further evaluation.

SHP2 is another upstream regulator of the RTK–SOS1–RAS network. RMC-4550 produced tumor shrinkage in neurofibroma models, but simultaneous MEK–SHP2 inhibition did not further increase response rates or the magnitude of tumor regression, and pERK suppression was not clearly greater than that produced by SHP2 inhibition alone (52). These findings suggest pharmacodynamic redundancy rather than consistent complementarity. Simultaneous MEK–SHP2 inhibition without biomarker selection therefore currently has relatively low translational priority. Patient selection, temporal targeting, or sequential treatment may warrant greater attention (49).

Cabozantinib provides a distinct strategy for upstream control. It inhibits MET, AXL, VEGFR2, KIT, and other kinases, while both cabozantinib and selumetinib have independently shown single-agent activity in PNs (34, 96). NCT06502171 is a registered phase I study of reduced-dose cabozantinib combined with selumetinib that is currently recruiting. Its clinical development is therefore more advanced than that of most candidate combinations, but no human combination efficacy data are yet available. Its translational value will first depend on whether clinically relevant exposure to both agents can be sustained within an acceptable long-term safety range rather than on the assumption that two active drugs will necessarily produce additive benefit.

MEK–SOS1 inhibition has comparatively clear PN-specific evidence of benefit over MEK monotherapy. KRAS–MEK inhibition is also promising, although its selumetinib comparator included historical data. Selumetinib plus cabozantinib has reached clinical dose exploration, whereas concurrent MEK–SHP2 inhibition requires stronger mechanistic justification (49, 52, 53, 114).

### 5.3 Targeting parallel survival pathways: compensatory signaling and adaptive survival

PI3K–AKT–mTOR and RAF–MEK–ERK share upstream RAS input but regulate partly distinct processes related to metabolism, growth, and survival. Prolonged MEK inhibition in three-dimensional PN cultures can increase compensatory phosphorylation of AKT and MEK, providing disease-relevant support for parallel pathway blockade (112).

In principle, MEK inhibition suppresses the principal proliferative driver, whereas inhibition of AKT or mTOR could reduce survival of residual cells. However, current evidence does not support unselected use of this strategy. Sirolimus and everolimus rarely induce substantial PN shrinkage as monotherapy, suggesting that mTORC1 dependence alone is insufficient for consistent tumor regression (101–104). Moreover, mTORC1 inhibition may relieve upstream feedback, and combining mTOR and MEK inhibition may increase chronic toxicities, including stomatitis, metabolic abnormalities, infection, and myelosuppression.

Selumetinib plus sirolimus also failed to produce the expected objective responses in a phase II MPNST study (113). MPNSTs cannot be used as direct surrogates for benign PNs, but this result reinforces the principle that simultaneous coverage of two biologically related pathways does not necessarily increase therapeutic efficacy.

MEK plus AKT/mTOR inhibition is therefore better considered a biomarker-selected investigational strategy than a general option for all patients with inadequate MEK-inhibitor responses. Its current translational priority is lower than that of MEK–SOS1 inhibition, for which PN-specific *in vivo* benefit over MEK monotherapy has been demonstrated. If treatment-induced AKT or mTOR dependence can be confirmed in human PNs, this strategy could be reassessed (36, 112).

### 5.4 Targeting residual tumor cells: cell-cycle and adaptive cell-state vulnerabilities

CDK inhibition may further restrict PN cells that remain viable or adopt an adaptive proliferative state during MEK inhibition. In PN cells resistant to TAK-733, transcriptomic and pharmacological screening identified upregulation of CDK1-related programs. Dinaciclib combined with TAK-733 more strongly inhibited cell viability, tumorsphere formation, and clonogenic growth than either agent alone and improved tumor control in patient-derived PN xenograft models (55).

A separate integrated transcriptomic and kinomic study identified concurrent CDK4/6 and MAPK activation in murine and human PNs. Abemaciclib combined with the ERK1/2 inhibitor LY3214996 enhanced antitumor activity in Schwann-cell and PostnCre mouse models (56). Compared with broad CDK inhibition, selective CDK4/6 targeting may be more attractive for prolonged treatment, although direct comparison with clinically established MEK inhibitors remains necessary. MAPK–CDK targeting can therefore be considered an intermediate-to-high-priority preclinical strategy that requires optimization of drug selection and dosing. Cell-cycle alterations reported during atypical progression provide indirect context but do not establish combination efficacy in benign PNs (59).

The Hippo–YAP/TAZ–TEAD axis may represent another adaptive state induced by MEK inhibition. Selumetinib increased YAP nuclear localization and transcriptional activity in PN models, whereas verteporfin plus selumetinib produced stronger inhibition in cellular and xenograft systems (57). These data support YAP–TEAD signaling as a potential combination target. However, verteporfin has multiple nonspecific effects and is not an ideal long-term systemic YAP inhibitor. Near-term development should therefore focus on pharmacologically selective TEAD inhibitors and confirmation of the corresponding adaptive transcriptional state in human PNs. The limited model and pharmacological evidence supports an exploratory priority below that of better-validated PN combinations (45, 57).

HSP90 inhibition may destabilize multiple signaling client proteins simultaneously. SNX-2112 and retaspimycin enhanced selumetinib activity in PN cell assays; the *in vivo* combination used SNX-2112 (58). The mouse experiment included five animals per group and lacked an HSP90-inhibitor-alone arm, limiting conclusions about *in vivo* synergy. However, the broad client-protein profile of HSP90 inhibition is associated with potential ocular, hepatic, and gastrointestinal toxicities, creating a high threshold for chronic use in a predominantly benign tumor.

FTY720 can modulate PP2A activity while also exerting immune effects through sphingosine-1-phosphate receptor 1 (S1P1) (54). Although some *in vitro* effects were enhanced by combination treatment, the *in vivo* combination did not clearly outperform both monotherapies, and PP2A-mediated effects could not be separated from S1P1-related effects. PP2A modulation therefore remains an early exploratory approach and currently has lower translational priority than CDK-based strategies supported by clearer PN-specific *in vivo* combination effects.

### 5.5 Targeting the tumor microenvironment: myeloid and stromal support

Microenvironment-directed combinations aim to suppress *NF1-deficient* Schwann cells while simultaneously reducing survival support from myeloid or stromal compartments. Complement C5 cleavage generates C5a, which regulates myeloid-cell recruitment and function through C5aR1/2. C5a is increased in PNs, and MEK inhibition can increase C5aR1 expression on macrophages. Short-term C5aR antagonism produced marked microenvironmental changes, but the combination did not establish clearly superior or durable volumetric control over MEK inhibition alone. Although some immunological and cytokine alterations persisted after treatment withdrawal, tumor growth resumed (66).

This study has important translational implications. Persistent histological or immunological changes cannot substitute for sustained tumor control. MEK–C5aR inhibition should therefore not currently be prioritized as a combination intended primarily to improve volumetric response. If the strategy is pursued, endpoints related to pain, inflammation, or post-treatment disease behavior may be more appropriate (66).

CSF1R inhibition can alter the myeloid compartment by affecting monocyte recruitment and macrophage survival. In a PN mouse model, treatment with the dual KIT/CSF1R inhibitor PLX3397 before tumor formation increased tumor volume compared with controls. In mice with established tumors, regression occurred in 4 of 14 treated animals and was associated with marked macrophage depletion. However, the overall difference in tumor response between treatment and control groups was not statistically significant (64). Pexidartinib is also associated with clinically important hepatotoxicity, and PN-specific benefit has not been established. MEK–CSF1R combination treatment therefore remains a low-priority exploratory strategy. Fibroblasts and ECM can provide nonimmune protection. TGF-β-driven basement membrane deposition alters tissue mechanics, whereas fibroblast-derived signals and P-glycoprotein may decrease intracellular drug exposure (70, 71). These observations provide a biological rationale for targeting stromal mechanisms. However, TGF-β signaling, integrin and focal-adhesion pathways, and drug-efflux systems are also widely involved in normal tissue homeostasis, creating substantial concerns regarding chronic systemic inhibition (73, 74).

The RXR agonist MSU-42011 reduced pERK and modified selected immune-cell populations in *NF1-deficient* cell systems and a syngeneic MPNST model (110); these findings cannot be directly extrapolated to benign human PNs. At present, microenvironment-directed strategies are therefore more valuable for mechanistic studies and biomarker development than as near-term priorities for clinical combination therapy.

## 6 Clinical translation of drug therapy for NF1-associated plexiform neurofibromas

Future drug development for NF1-associated PNs should not aim to move all patients from MEK-inhibitor monotherapy to combination treatment. Observation remains appropriate for stable, mildly symptomatic lesions, whereas persistent growth, inability to achieve complete resection, progressive pain, or threatened major function provide stronger indications for systemic therapy (2, 42). For patients who achieve sustained symptom improvement and disease control with MEK inhibition, there is currently no evidence supporting routine addition of a second drug. In contrast, patients who derive little benefit despite adequate exposure, experience progression during ongoing therapy, or cannot maintain an effective dose because of toxicity are more appropriate candidates for studies of mechanism-guided treatment modification. Representative registered trials addressing these translational questions are summarized in **Table 3**.

**Table 3:** Registered interventional studies relevant to NF1-associated plexiform neurofibromas by trial phase, with estimated enrollment, dosing, primary endpoints, and planned assessment windows (records checked October 12, 2026).

Phase and registration	Estimated enrollment and population	Investigational oral regimen	Registered primary endpoint	Planned assessment window	Registry status
Phase I NCT02390752	54 total; age 3–35 y Basket study including NF1-PN	Pexidartinib orally daily; dose not specified in public record; 28-day cycles	RP2D selection based on safety/tolerability	First cycle	Recruiting
Phase I NCT06502171	30; age ≥16 y Progressive/symptomatic NF1-PN	Cabozantinib starts at 20 mg QD plus selumetinib dose escalation	DLTs and overall toxicity profile	DLT window 16 wk; toxicity assessment 12 mo	Recruiting
Phase I NCT06961565	56 adults Symptomatic, inoperable/incompletely resected/recurrent PN	PAS-004 4, 8, 12 or 18 mg QD (escalation); 2 expansion doses selected from Part A	Safety/tolerability, including DLTs and AEs	Part A: cycle 1; Part B: cycle 6 (28-day cycles)	Recruiting
Phase II NCT05331105	70; age 18–80 y Symptomatic, inoperable NF1-PN	Tunlametinib 9 mg BID in IIa (dose modification allowed); recommended regimen in IIb	Confirmed ORR by volumetric MRI (REiNS)	Cycles 4–32 every 4 cycles, then every 8 cycles (21-day cycles)	Listed as recruiting
Phase II NCT06188741	200 across study parts; age 1–8 y Screening and high-risk asymptomatic PN	Selumetinib 25 mg/m² BID vs observation; subsequent intermittent-dosing evaluation	PFS for selumetinib vs observation	60 mo	Recruiting
Phase II NCT06541847	25; mainly age ≥16 y, plus age 12–15 y cohort Progressive/symptomatic PN	HLX-1502 orally TID; dose not specified; 12 cycles, extendable to 24 (28-day cycles)	PN response rate by blinded central volumetric MRI (PR ≥20% decrease)	12 mo (primary); 24 mo (secondary)	Recruiting
Phase III NCT07407803	177 adults Symptomatic, inoperable NF1-PN	TQ-B3234 50 mg QD vs placebo; 28-day cycles	IRC-assessed ORR (REiNS)	Timing inconsistent within registry	Recruiting

Source: ClinicalTrials.gov, individual records identified by NCT number (https://clinicaltrials.gov/), accessed October 12, 2026. AE, adverse event; BID, twice daily; DLT, dose-limiting toxicity; IRC, independent review committee; mo, months; ORR, objective response rate; PFS, progression-free survival; PN, plexiform neurofibroma; PR, partial response; QD/TID, once/three times daily; REiNS, Response Evaluation in Neurofibromatosis and Schwannomatosis; RP2D, recommended phase II dose; wk, weeks; y, years.

Phase colors: pale blue, phase I; pale amber, phase II; pale purple, phase III. Colors indicate trial phase only and do not denote efficacy, evidence quality, or recruitment status.

Future treatment should also extend beyond simultaneous two-drug therapy. Mirdametinib retains clinical activity with a 3-week-on/1-week-off schedule, whereas TRAM-01 showed that some patients required further intervention relatively soon after completion of a fixed treatment course (38, 39). These findings support further evaluation of continuous treatment, intermittent schedules, and rechallenge after treatment withdrawal. For a tumor that previously remained sensitive to MEK inhibition and regrows only after discontinuation, rechallenge may be more evidence-based than immediate addition of a second target. In patients limited primarily by toxicity, switching MEK inhibitors or modifying the dosing schedule should also remain important options (40, 41, 78).

More informative biomarkers are needed to guide therapeutic selection. Human selumetinib studies have demonstrated substantial pERK suppression, whereas consistent compensatory AKT activation was not observed at all biopsy time points (36). Parallel-pathway combinations should therefore not be selected solely on the basis of *in vitro* signaling observations. Future studies could integrate drug exposure with RAS-GTP or RTK activity, treatment-induced pAKT changes, YAP/TEAD transcriptional states, and microenvironmental features to distinguish different causes of inadequate response. Because repeated biopsies of PNs are constrained by neural anatomy and spatial heterogeneity, paired tissue studies could be incorporated into clinically indicated surgical procedures and combined with imaging and pharmacokinetic measurements (62).

No molecular biomarker is currently validated to select a particular MEK inhibitor or combination. Volumetric MRI and patient-reported outcomes monitor treatment benefit, whereas tissue pERK primarily demonstrates target engagement (34, 36, 76). Candidate resistance markers require longitudinal association with progression during adequate drug exposure before clinical use (36, 55, 57, 112).

Systemic therapy should also remain integrated with surgery. Pharmacological tumor shrinkage may alter the anatomical relationship of a PN to critical nerves or blood vessels and thereby improve resectability (2, 42). For potentially operable lesions, indefinite drug therapy should not replace periodic reassessment of surgical feasibility, anticipated functional morbidity, and perioperative management. Perioperative safety will be particularly relevant for combinations involving antiangiogenic drugs or pathways that affect wound healing (4, 5, 22, 23, 29, 32, 33).

The predominantly benign and chronic nature of NF1-associated PNs also argues against direct adoption of maximal treatment-intensity principles used for metastatic malignancies. Long-term selumetinib follow-up demonstrates that some toxicities emerge only after prolonged exposure, and discontinuation rates differ among MEK inhibitors (35, 90). A 2026 preclinical analysis and clinical case series found no evidence that selumetinib accelerated MPNST development; four of 99 trial participants developed MPNST during or shortly after treatment, and all had recognized high-risk features. These observations do not establish causality and support continued surveillance for malignant transformation during MEK inhibition (115). Future trials should therefore evaluate not only ORR but also pain, function, delivered dose intensity, treatment interruptions, and long-term safety. For combination therapy, additional efficacy is clinically meaningful only when it outweighs the added toxicity and treatment burden. Volumetric MRI remains essential, but patient-reported outcomes and lesion-specific functional measures should contribute equally to assessments of therapeutic value (26–29, 34, 37, 38, 40, 41, 76, 78, 82).

## 7 Discussion

MEK inhibitors have established the current foundation of systemic therapy for NF1-associated PNs. Studies in children and adults consistently show that suppression of RAS–MAPK signaling can produce clinically meaningful volumetric tumor reduction and, in some patients, improvements in pain and function (34, 36–38). ORRs across individual MEK inhibitors should not be ranked directly because response-assessment windows, follow-up duration, and eligibility criteria vary considerably. More consistent across studies are the rarity of complete responses, persistence of residual tumor, and frequent dependence of long-term disease control on continued treatment exposure (35, 39).

These limitations provide a rationale for studying combination therapy but do not justify routine multidrug treatment. Among current approaches, combined MEK–SOS1 inhibition produced greater antitumor effects than MEK monotherapy in a PN-specific mouse model and therefore has relatively strong preclinical translational potential (53). KRAS–MEK inhibition provides a further preclinical option, with greater tumor regression than KRAS inhibition alone (114). Its incremental benefit over MEK monotherapy requires confirmation with a fully concurrent comparator. Selumetinib plus cabozantinib is being evaluated in a phase I dose-exploration study. Whether the combination provides net clinical benefit beyond its two active components remains unknown. CDK- and YAP/TEAD-directed combinations are supported by disease-relevant preclinical findings and now require improved pharmacological agents and confirmation of target dependence in human PNs (55–57).

Negative findings are equally important for prioritizing development. Simultaneous MEK–SHP2 inhibition did not demonstrate clear incremental activity, C5aR targeting altered the tumor microenvironment without preventing renewed tumor growth, and MEK–mTOR approaches are constrained by weak tumor-shrinking activity of mTOR monotherapy and the potential for overlapping toxicity (52, 66, 101–104, 113). These observations emphasize that mechanistic connectivity between signaling pathways is not sufficient to establish a rational combination. In a tumor requiring prolonged therapy, the second agent should address a clearly defined limitation of monotherapy and should demonstrate incremental activity over an effective single-agent comparator in disease-relevant models.

Current evidence for combination therapy remains predominantly preclinical and is derived from cell systems, patient-derived xenografts, and genetically engineered animal models. None fully reproduces the prolonged natural history, complex neural anatomy, and low tolerance for chronic toxicity that characterize human PNs. The status of ongoing clinical trials continues to evolve; registration information does not establish treatment efficacy. Preclinical findings should therefore not be presented as established treatment strategies, and unpublished or incompletely published evidence should be carefully evaluated (2, 9, 17–20, 42, 53, 55–58, 61–63, 66).

Overall, the next advance in NF1-associated PN therapy is unlikely to arise from indiscriminate escalation of treatment intensity. Greater progress is more likely to result from improved patient selection and closer alignment between therapeutic mechanisms and specific patterns of treatment failure. Optimization of MEK-inhibitor monotherapy remains important for many patients who achieve sustained benefit. Rational combinations, sequential treatment, or alternative dosing strategies should primarily be investigated in patients with clearly defined pharmacodynamic insufficiency, progression during adequate treatment, or dose-limiting toxicity. MEK–SOS1 inhibition, selumetinib plus cabozantinib, and selected approaches targeting residual tumor-cell states remain priorities for further study. KRAS–MEK inhibition adds a candidate that warrants confirmation against MEK monotherapy. Negative combination data should guide de-prioritization of strategies unlikely to provide meaningful incremental benefit (35, 39, 52, 53, 55–58, 66, 90, 114).

The future treatment paradigm for NF1-associated PNs may therefore evolve from fixed monotherapy toward a mechanism-guided, dynamically adjusted therapeutic pathway rather than simply from one drug to permanent dual therapy. A new strategy should ultimately be judged by whether it provides clinically meaningful benefit beyond an active monotherapy within a sustainable long-term safety window. Tumor volume, pain, function, resectability, and disease control after treatment withdrawal should collectively determine its therapeutic value (2, 34–39, 42, 76). Combination trials should use an active MEK-inhibitor comparator when feasible and account for age, prior treatment, and delivered dose intensity. Follow-up after withdrawal is needed to distinguish on-treatment benefit from durable treatment-free control (34–39, 42, 76, 78).
